# Primary Hyperparathyroidism in Pregnancy: A Two-Case Report and Literature Review

**DOI:** 10.1155/2015/171828

**Published:** 2015-03-29

**Authors:** A. D. Herrera-Martínez, R. Bahamondes-Opazo, R. Palomares-Ortega, C. Muñoz-Jiménez, M. A. Gálvez-Moreno, J. M. Quesada Gómez

**Affiliations:** Department of Endocrinology and Nutrition, Reina Sofia University Hospital, Avenida Menéndez Pidal, s/n, 14004 Córdoba, Spain

## Abstract

Primary hyperparathyroidism (PHPT) in pregnant women is an uncommon disease. It could be easily misdiagnosed because of physiologic changes during pregnancy; in some cases, patients could remain asymptomatic maintaining elevated calcium serum levels, and this situation represents a threat to the health of both mother and fetus. We present two cases of PHPT during pregnancy and their evolution after surgical treatment in the second trimester; there were no observed complications during pregnancy or delivery in our patients. Early diagnosis and medical/surgical treatment in PHPT are necessary for avoiding maternal and fetal complications which could not be predicted based on duration or severity of hypercalcemia. An appropriate management of PHPT during pregnancy is necessary for preserving the health of both the woman and the fetus.

## 1. Introduction

Primary hyperparathyroidism (PHPT) results from inappropriate overproduction of parathyroid hormone from one or many parathyroid gland(s) [1]. It is the third most common endocrine disorder (after diabetes and thyroid disease), and the most common cause of hypercalcemia. It affects 0.3% of the general population, with a twice higher incidence in women [2], and a similar incidence in pregnant and nonpregnant women [3]. PHPT consists in the presence of elevated serum calcium, low or low-normal phosphate with elevated or inappropriately normal PTH with hypercalciuria (based on urine calcium clearance/creatinine clearance ratio). PHPT usually occurs as the result of sporadic parathyroid adenomas or carcinomas, and it can also be seen in association with multiple endocrine neoplasias and in rare genetic syndromes or metabolic diseases [1].

PHPT during pregnancy is an uncommon disease that could be misdiagnosed; it represents a threat to the health of the mother and fetus. Physiological changes of pregnancy may attenuate PHPT signs and symptoms: hypoalbuminemia, calcium transport across the placenta, and an increased glomerular filtration rate contribute to the appearance of lower calcium levels. At the same time, parathyroid hormone- (PTH-) mediated bone resorption is inhibited by estrogens, which causes a dose-related reduction in serum calcium in pregnant patients [3].

Most common symptoms in women are nephrolithiasis, hyperemesis, pancreatitis, or hypercalcemic crisis. Maternal complications have been reported even in 67% of patients [4], and untreated disease could commonly complicate fetal development and mortality. It is not possible to predict complications based on the duration of symptoms or serum calcium levels.

Early diagnosis and medical and/or surgical treatment should be evaluated for avoiding complications. We present two cases of PHPT during pregnancy and their evolution after surgical treatment.

## 2. Case 1

A 31-year-old Caucasian woman was referred to our hospital during the second trimester of pregnancy (22 weeks) for evaluation of hypercalcemia. There was no history of diseases. Her family history did not reveal any case of hypercalcemia or endocrine tumors. Her medications during pregnancy included iodine and folic acid supplements daily. The patient was asymptomatic, keeping normal blood pressure. In her routine analysis, an adjusted serum calcium level of 11.2 mg/dL (reference range: 8.5–10.5 mg/dL) was determined. New determinations were reported: phosphate 1.9 mg/dL (reference range: 2.5–4.9 mg/dL), PTH 132.24 pg/mL (reference range: 11–54 pg/mL determined by chemiluminescence assays), urine calcium clearance/creatinine clearance ratio 436 mg/mg (reference range: 0.1–0.3 mg/mg), and 25-hydroxy vitamin D (25OH-D) 31.21 ng/mL (reference range: 20–80 ng/mL). A neck ultrasound showed an 8 × 6 × 3 mm solid hypervascular nodule in the lower left pole of the thyroid gland, and this lesion was compatible with a parathyroid adenoma. The patient had surgery in the 24th week of pregnancy, and an adenomectomy was performed, conserving the other parathyroid glands. The patient had an immediate normalization of serum calcium levels, 9.3 mg/dL; phosphate, 3.8 mg/dL; PTH, 20.4 pg/mL; and 25-OH.-D, 66.38 ng/mL. Any other incidence during pregnancy was reported. A female newborn was delivered, with normal weight and height, and blood tests showed normal serum values of calcium. During the following, both mother and newborn did not develop complications or changes in their calcium or phosphate levels.

## 3. Case 2

A 31-year-old woman was first evaluated in our hospital during the 11th week of pregnancy due to subclinical hypothyroidism (TSH 5.27 mU/L; T4L: 1.18 ngmL). She had a past history of nephrolithiasis and renal colic. Her family history did not reveal any case of hypercalcemia or endocrine tumors. Her medications during pregnancy included iodine and folic acid supplements daily, associated with treatment with 50 mcg of levothyroxine. The patient was asymptomatic, and a serum analysis during a renal colic determined an adjusted serum calcium level of 12.3 mg/dL. New determinations were reported: adjusted serum calcium, 12.7 mg/dL; phosphate, 2.1 mg/dL; PTH, 95.8 pg/mL; urine calcium clearance/creatinine clearance ratio, 0.26 mg/mg; 25-OH.-D, 31.21 ng/mL. A neck ultrasound revealed a 9 × 7 × 5 mm solid hypervascular nodule in the middle left pole of the thyroid gland, and this image was compatible with an intrathyroid parathyroid adenoma. This patient went to surgery during the 21st week of pregnancy, and an adenomectomy was performed, conserving the other parathyroid glands. Blood tests after surgery revealed the following: serum calcium, 9.2 mg/dL; phosphate, 3.8 mg/dL; PTH, 16.1 pg/mL; and 25-OH.-D, 28.4 ng/mL. After the surgery the patient required increasing doses of thyroid hormone adjusted to pregnancy evolution. During the third trimester of pregnancy, pregnancy-diabetes was diagnosed, and it was controlled with diet and life style modifications. No hypertension was developed during pregnancy. The patient underwent a normal delivery without complications, the newborn had normal serum levels of calcium and phosphate, and his weight and height were also normal. After birth, the patient had normal serum levels of calcium, phosphate, PTH, and TSH. The patient and her newborn have kept asymptomatic, maintaining normal values of serum calcium metabolism markers.

## 4. Discussion

PHPT results from inappropriate overproduction of parathyroid hormone from one or many parathyroid glands and presents with hypercalcemia. Precise regulation of extracellular and intracellular calcium is essential for normal physiological processes such as cell signaling, neural function, muscular function, cardiac contractility, hormone release regulation, and bone metabolism. Parathyroid hormone increases receptor-mediated tubular reabsorption of calcium in the kidney, stimulates release of skeletal calcium stores, upregulates 1-*α*-hydroxylase leading to increased 1,25-dihydroxy-vitamin D production, and increases calcium reabsorption from the gastrointestinal tract [5]. During pregnancy, elevated levels result in fetal PTH suppression which accounts for the hypocalcemic effects on the fetus at birth [6].

The association between PHPT and pregnancy is uncommon; mostly pregnant women remain asymptomatic even with high serum calcium. Cases reports reveal that maternal and fetal complications could not be predicted based on duration or severity of hypercalcemia [7]. Maternal complications include nephrolithiasis (24–36%), radiographic bone disease (13–19%), pancreatitis (7–13%), hyperemesis gravidarum, muscle weakness, confusion, and hypercalcemic crisis. The Norman Parathyroid Clinic in Florida has reported calcium levels >11.4 mg/dL in association with higher levels of fetal loss in 72% of their cases [8]. In addition, there is concern for further increase in calcium levels postpartum when fetal shunting is removed [9]. Other clinical findings include maternal hypertension, insulin resistance, and endothelial damage [4].

Fetal complications in untreated PHPT mothers can affect 80% of newborns, and 27–31% of those complications are identified as neonatal death. Other complications include intrauterine growth restriction, low birth weight, preterm delivery, and intrauterine fetal demise. Up to 50% of neonates experience postpartum hypocalcemia secondary to elevated maternal calcium levels that suppress the fetal parathyroid glands, which mostly is a transient phenomenon. However, some cases have been reported in which the neonatal hypoparathyroidism lasts up to several months or is permanent [10].

For early diagnosis, a minimally invasive approach with ultrasound is best suited to mitigating risk to mother and fetus. Most patients with PHPT have a single adenoma (~80% of cases), but multigland disease can occur in 10–15% of cases and double adenomas in 4-5%, and parathyroid carcinoma is a rare cause (<1%) of hyperparathyroidism [11, 12].

The only definite cure for PHPT due to parathyroid adenomas is the surgical resection [1]. In 2008, the Third International Workshop on Asymptomatic Primary Hyperparathyroidism revised the indications for surgery in asymptomatic patients, and these include age less than 50 years, serum calcium 0.25 mmol/L above the upper limit of normal, creatinine clearance <60 mL/min, DXA *t*-score <−2.5 at any site, and/or previous fragility fracture [13]. Surgery is also the preferred option for management of PHPT in pregnant women, children, and adolescents [1, 7].

Conservative medical therapy should be considered in asymptomatic patients with a calcium level of <11 mg/dL [4]. Medical treatment includes oral hydration and low-calcium diet, with or without forced diuresis with a low dose of loop diuretic, considering risk and benefits of diuretics during pregnancy. Phosphate therapy (1.5–3 g/day) has a substantial risk of calcium phosphate precipitation; for this reason it is not often recommended [10]. Subcutaneous calcitonin could represent a treatment option, and it has a category C rating, with a limited efficacy due to tachyphylaxis. Cinacalcet has also been used in conjunction with calcitonin, demonstrating highly effective reduction in PTH levels [14]. Cinacalcet has a category C rating, but studies remain lacking to evaluate effects on mother and fetus/neonate [15]. Regular evaluation of the fetus is always recommended [14].

As PHPT represents a significant risk for maternal and fetal complications, surgical treatment should be considered early in pregnant patients [7]. Other authors consider surgery in the second trimester after failing of medical treatment [9]. There have not been previously reported operative complications or fetal loss related to surgery during pregnancy. In our patients, both cases were diagnosed in the second trimester of pregnancy; based on the available evidence, surgery was suggested as first therapeutic option, without complications during their evolution [7, 9].
